# Fast and broadband spatial-photoresistance modulation in graphene–silicon heterojunctions

**DOI:** 10.1515/nanoph-2024-0084

**Published:** 2024-06-17

**Authors:** Ruxia Du, Wenhui Wang, Huiwen Lin, Xinlei Zhang, Hao Wu, Beibei Zhu, Xu Jing, Xing Gu, Zhenhua Ni, Li Tao

**Affiliations:** School of Materials Science and Engineering, 12579 Southeast University, Nanjing 211189, P.R. China; Key Laboratory of Quantum Materials and Devices of Ministry of Education, School of Physics, 12579 Southeast University, Nanjing 211189, P.R. China; Key Laboratory of Quantum Materials and Devices of Ministry of Education, School of Materials Science and Engineering, Nanjing, P.R. China

**Keywords:** graphene, bipolar resistance effect, position, near infrared, plasma

## Abstract

Different types of devices with modulable resistance are attractive for the significant potential applications such as sensors, information storage, computation, etc. Although extensive research has been reported on resistance effects, there is still a need for exploring new mechanisms that offer advantages of low power consumption, high sensitivity, and long-term stability. Here, we report a graphene–Si based spatial-dependence photo-rheostat (SDPR), which enables bipolar resistance modulation in the range of 5 mm with a resistance sensitivity exceeding 1,000 Ω/mm at operating wavelengths from visible to near infrared band (1,550 nm). Especially, at ultra-low energy consumption, the device can achieve modulation of even 5 orders of magnitude of resistance and response speed up to 10 kHz. A theoretical model based on carrier dynamics is established to reveal the diffusion and drift of carriers as a mechanism explaining such experimental phenomenon. This work provides a new avenue to modulate resistance at low power consumption as novel opto-potentiometers in various photoelectric applications.

## 1 Introduction

Resistance is the property reflecting the transport behavior of carriers or ions, and its manipulation is an old and intriguing topic that has attracted great interest for its multiple functions. To date, some materials and devices can show the ability of achieving two or more stable resistance states. Various schemes for modulating resistance have been demonstrated by utilizing different stimulus strategies that are based on unique physical mechanisms. Some notable examples include the superconducting effect at super low-temperature [1], [2], [3], [4], [5], [6], giant magnetoresistance effect (GMR) in very weak in-plane magnetic field [7], [8], [9], [10], [11], [12], the memristor effect aided by the formation of conductive filaments or interface barriers manipulation [13], [14], [15], [16], and the reversible changes of high resistive state (HRS) and low resistance state (LRS) in crystalline and amorphous phase transition materials [17]. Other methods such as light stimulation can also be utilized to regulate resistance through operating laser power based on the photo-resistance effect. Besides, under nonuniform irradiation, the distribution of photogenerated carrier concentration can be regulated by using the spatial movement of the laser, thus producing lateral photoelectric effect [18] and photo-induced bipolar-resistance effect (BRE) [19], [20], [21], [22], [23]. The former is known for the use in position-sensitive detectors (PSD), and various structures based on 2D materials have been reported in last decade [24], [25], [26], [27], [28], [29], [30], [31], [32], [33]. Whereas the latter is considered to be one effective method to vary the resistance of the device. Until now, the BRE has been discovered in MOS structure [21], [22], [23], CIGS heterojunction [20], and other structures. However, BRE devices with broadband, fast speed, and low power consumption are still desired, and the internal mechanism needs to be further investigated.

In this article, we report the observation of spatial-modulated lateral resistance effect in large-area graphene–Si under nonuniform illumination. The SDPR shows multi-valued linear resistance as well as high-low resistance outputs at the operating wavelength covering the visible-near infrared band (1,550 nm). In the linear modulation region, the resistance sensitivity of pristine graphene-based device can be up to 240 Ω/mm, which can be further improved to 1,263 Ω/mm by plasma modulation. By using BRE, the resistance modulation range can even span 5 orders of magnitude at very low power consumption. Also, the switching of HRS and LRS can support up to 10 kHz AC voltage pulse signal. With the help of an analytical theoretical model, the mechanism of the position-sensitive rheostat is explained based on the diffusion and drift model of carriers. This ultra-fast, highly sensitivity, broadband, position-dominated photo-rheostat with low power consumption has promising potential in photoelectric resistors, switches, and versatile photoelectric devices.

## 2 Results and discussion

**Figure 1:**
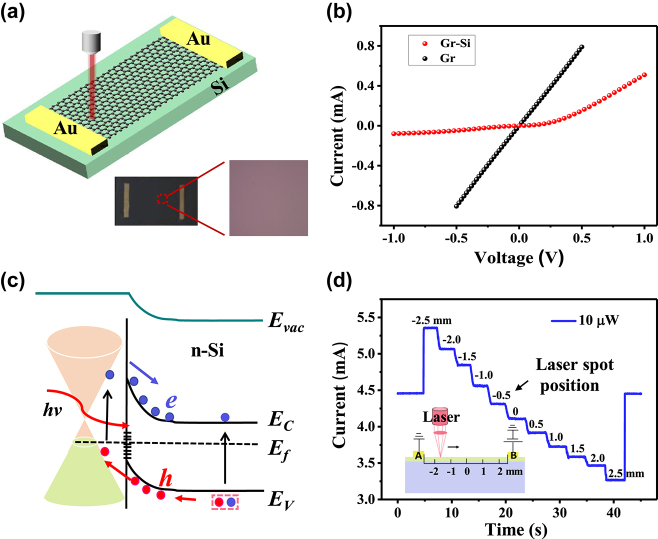
Band structure and electrical characteristics of the device. (a) Schematic illustration of the graphene–Si heterojunction device and the optical microscopy photograph of the device. (b) I–V curve of graphene on the Si substrate. The inset is IV curve of graphene–Si heterojunction. (c) Energy band diagrams of the heterojunction. Due to the surface state of Si, there is a built-in electric field at the interface pointing from Si to graphene. (d) The current of device with 633 nm laser irradiated at different positions, *V* = 4 mV, *P* = 10 μW. The inset is schematic diagram of device operation.

Figure 1a displays the schematic of graphene–Si hybrid SDPR. Lightly *n*-doped silicon (*ρ* = 1–10 Ω cm) was employed as the substrate for its relatively long carrier lifetime. The monolayer graphene grown by CVD was transferred to the silicon to construct the device. The Raman spectra of the graphene are shown in Figure S1a, which suggest that high-quality graphene was obtained due to the lack of any defect peaks [34]. From the linear I–V characteristics of the graphene on Si, it can be concluded that ohmic contacts are formed between graphene and Au electrodes, as shown in Figure 1b. A typical rectification behavior can be observed from I–V curve of graphene–Si heterojunction, indicating the presence of a built-in electric field directed from bulk Si to graphene at the interface. The surface state of Si is the main contributor to the energy band bending, which results in the generation of the built-in electric field [35], [36]. Under incident light irradiation, the build-in field can efficiently separate photo-induced electron–hole pairs, transferring the holes into graphene while drifting the electrons to the bulk Si, as shown in Figure 1c. Compared to silicon, the high carrier mobility of graphene allows holes to quickly diffuse laterally and then be collected by electrodes. Meanwhile, graphene also acts as a photosensitive layer, allowing for broadband response due to its zero-band gap energy structure, which will be discussed later. The photoresponse characteristics of the device were performed, as shown in Figure S1b, which shows high sensitivity and good stability. Figure 1d depicts the current variation of the device being illuminated at different locations. The response characteristics are recorded while a focused laser beam (spot size ∼2 μm) moving from electrode A (−2.5 mm) to electrode B (2.5 mm) (see Section 4). Clear and robust steps reveal that the device has high position resolution, even at a low incident power of 10 μW. The device works akin to a conventional sliding rheostat, except that the manipulation strategy is the irradiation position on the channel instead of mechanical slider, which demonstrates the potential of the device as a position-dependent photoresistance device.

**Figure 2:**
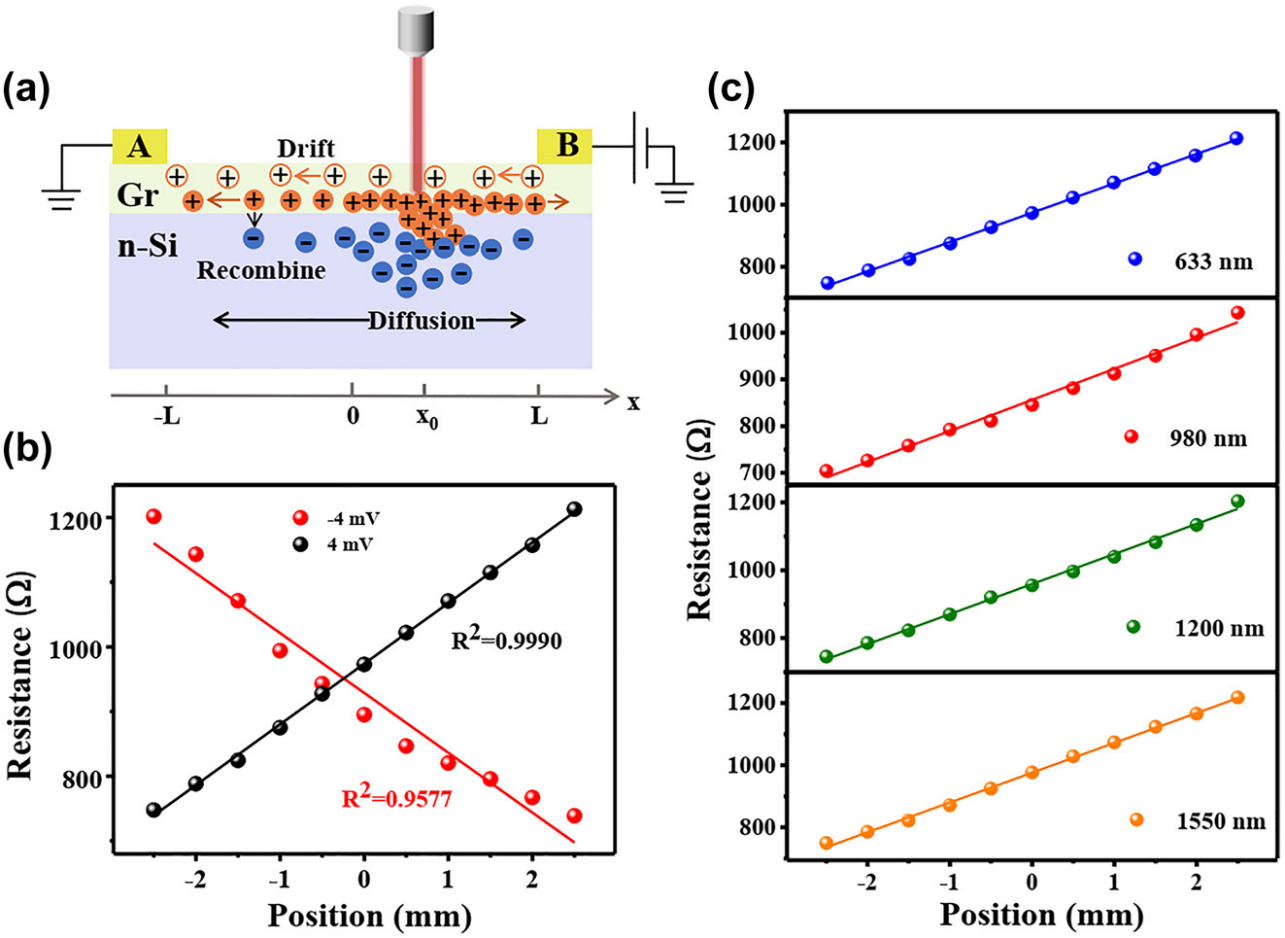
Position-dependent characteristics of devices at different voltages and wavelengths. (a) Carrier dynamics model in device operation. (b) Dependence of device resistance and laser spot position at ±4 mV. The solid lines are linear fitting curves (laser: 633 nm, 10 μW). (c) The position dependence of resistance under different laser wavelength of 633 nm, 980 nm, 1,200 nm and 1,550 nm.

To see further into the photo-induced electrical modulation phenomenon, the carrier dynamics and the position-dependent resistance of the device are investigated. As shown in Figure 2a, the electron–hole pairs generated under laser irradiation spontaneously diffuse laterally after being separated by the built-in electric field at the interface. This interacts with the drift current driven by the bias voltage, resulting in the position-dependent characteristics of the device resistance. The resistance of the device is extracted at different irradiation positions shown in Figure 2b. It can be seen that the resistance of the device correspondingly increases from 748 Ω to 1,212 Ω as the laser is moved from electrode A to electrode B. However, when a reversed bias voltage is applied to the device, the resistance decreases conversely from 1,201 Ω to 739 Ω, which is indicative of the photo-induced bipolar resistance effect characteristics [23]. Notably, the resistance exhibits a near relationship with laser spot position. The linear fit exhibits an *R* ^2^ close to be 1 (Figure 2b), which indicates the excellent linearity. The resistance sensitivity of the device in the linear operating range can reach ∼92 Ω/mm, which is appreciable for a device with a 5 mm channel length at such a low power consumption. Moreover, this sensitivity can be further improved by using surface engineering, which will be discussed in detail later. Owing to the co-absorption of Si and graphene, the device achieves a photoresponse that spans the visible and near infrared (Figure S2). Figure 2c shows the position-dependent characteristics of the resistance of the device under 633 nm, 980 nm, 1,200 nm, and 1,550 nm laser irradiation. All the examined wavelengths exhibit good linear relationships, which demonstrate the broadband potential of the SDPR.

As mentioned above, the drift carrier of the device is affected by the photogenerated carrier diffusion. The resistance evolution of the device at different bias voltages is shown in Figure S3, where the resistance sensitivity can be observed to decrease with the increase of the bias voltage. Generally, small bias leads to low average drift velocity of the intrinsic carrier. It is reasonable to speculate that there are interactions of the drift motion of intrinsic carriers and inhomogeneous diffusion of photocarriers. To further understand the physical mechanisms of the device, a model is established based on the carrier dynamics process.

When a laser with wavelength *λ* and power *p* (light intensity *I* = *p*/*S*_light_, where *S*_light_ is area of laser spot) is irradiated on the device, the photogenerated electron–hole pairs are generated simultaneously in graphene and bulk Si. When the equilibrium state is reached, the concentration of the photogenerated carrier is defined as Δ*p*(*λ*) = Δ*n*(*λ*) = *βαIτ*, where *β* is the quantum yield, *α* is the absorption coefficient, and *τ* is the nonequilibrium carrier lifetime. Under the action of the built-in electric field at the interface, the photogenerated electron–hole pair is separated, and the holes have a certain probability *C* to enter into the graphene layer, and also have a certain probability to recombine with the electrons in Si. The concentration of holes entering into the graphene is given by, Δ*p* = Δ*p*(*λ*)[1 − *C* ^*τp*/Δ*p*(*λ*)^]. These holes entering the graphene diffuse laterally (from the illumination region to the nonillumination region) and form a concentration gradient. According to the diffusion theory of carriers *D*_*p*_d^2^Δ*p*_0_(*x*)/d*x* ^2^ = Δ*p*_0_(*x*)/*τ*, in which *D*_*p*_ is the diffusion coefficient of hole in graphene, the hole concentration at *x* on both sides can be calculated as:

(1) $$\Delta p=\left\{\begin{array}{cc} \Delta p_{0}e^{-\frac{x-x_{0}}{\lambda_{G}}}\; & \left(x_{0}<x<L\right)\\ \Delta p_{0}e^{-\frac{x_{0}-x}{\lambda_{G}}}\; & \left(-L<x<x_{0}\right) \end{array}\right.$$

where *x*_0_ is the position of laser spot, *λ*_*G*_ is the diffusion length of hole in graphene ( $\lambda_{G}=\sqrt{D_{p}\tau }$ ). Since the concentration gradient of photogenerated holes on both sides of the laser spot is different, the lateral diffusion current cannot be completely balanced. If the laser spot *x*_0_ is closer to electrode B, more photo-induced holes will be collected by electrode B than by electrode A, resulting in the formation of a lateral electric field *ɛ*_*x*_ from B to A. This electric field will also drive the carrier drift motion, in the steady state, the drift current, and the diffusion current will reach a balance. Therefore, the electric intensity can be deduced as:

(2) $$p_{0}q\mu_{p}\varepsilon_{x}-qD_{p}\frac{\text{d}\Delta p_{0}{\left(x\right)}_{\text{left}}}{dx}-qD_{p}\frac{\text{d}\Delta p_{0}{\left(x\right)}_{\text{right}}}{dx}=0$$

For *p*-type graphene, the concentration of minority electrons is much smaller than that of hole concentration *p*_0_ >> *n*_0_. Hence, the drift motion of the electron can be considered to be negligible. The *V*_*x*_ induced by the photogenerated hole can be expressed as:

(3) $$V_{x}=\overset{L}{\underset{-L}{\int }}\varepsilon_{x}dx=\frac{D_{p}\Delta p_{0}}{p_{0}u_{p}}\left(e^{-\frac{L-x_{0}}{\lambda_{G}}}-e^{-\frac{x_{0}+L}{\lambda_{G}}}\right)$$

According to Einstein’s relation, *D*_*p*_/*μ*_*p*_ = *k*_0_ *T*, the *V*_*x*_ can be obtained

(4) $$V_{x}=\frac{k_{0}T\Delta p_{0}}{p_{0}q}\left(e^{-\frac{L-x_{0}}{\lambda_{G}}}-e^{-\frac{x_{0}+L}{\lambda_{G}}}\right)$$

where, the value range of *x*_0_ is −*L* < *x*_0_ < *L*, *k*_0_ is the Boltzmann constant, *T* is temperature, *p*_0_ is the concentration of intrinsic hole-carrier, and q is a constant. Therefore, it can be deduced that the *V*_*x*_ is positively correlated with *x*_0_ under fixed temperature and illumination. As the laser spot at the channel center (*x*_0_ = 0), the number of photogenerated holes reaching the two electrodes per unit time is the same, and the lateral *V*_*x*_ is 0, which is consistent with the experimental measurement. When the laser spot gets closer to the edges of the electrodes (*x*_0_ → −*L*, *L*), $\left|V_{x}\right|$ reaches a maximum, given by:


(5) $${\left|V_{x}\right|}_{\max}\approx \left|V\left(\pm L\right)\right|=\left|\frac{k_{0}T\Delta p_{0}}{p_{0}q}\left(1-e^{-\frac{2L}{\lambda_{G}}}\right)\right|$$

As the resistance measured, a set of voltages *V*_bias_ with positive and negative polarities are applied, causing the carriers to drift in the corresponding direction. In this scenario, two modes of carrier movement exist under illimitation, namely, drift and diffusion. Then the relation between the resistance and *V*_bias_ at laser position *x*_0_ can be obtained as:

(6) $$R=\frac{V_{\text{bias}}R_{0}}{V_{\text{bias}}-\frac{k_{0}T\Delta p_{0}}{p_{0}q}\left(e^{-\frac{L-x_{0}}{\lambda_{G}}}-e^{-\frac{x_{0}+L}{\lambda_{G}}}\right)}$$

In which *R*_0_ is the original resistance of the graphene between A and B electrodes in the absence of illumination. Equation (6) suggests that the lateral resistance is a function of the voltage *V*_bias_ and laser spot position *x*_0_ under certain temperature with a fixed light intensity. When the polarity of *V*_bias_ is different, *R* shows the opposite monotonicity and presents obvious symmetry, which is consistent with the change of bipolar resistance shown by the device. The developed model has been shown to be suitable for aiding the understanding of the physical mechanisms governing the intriguing BRE feature.

**Figure 3:**
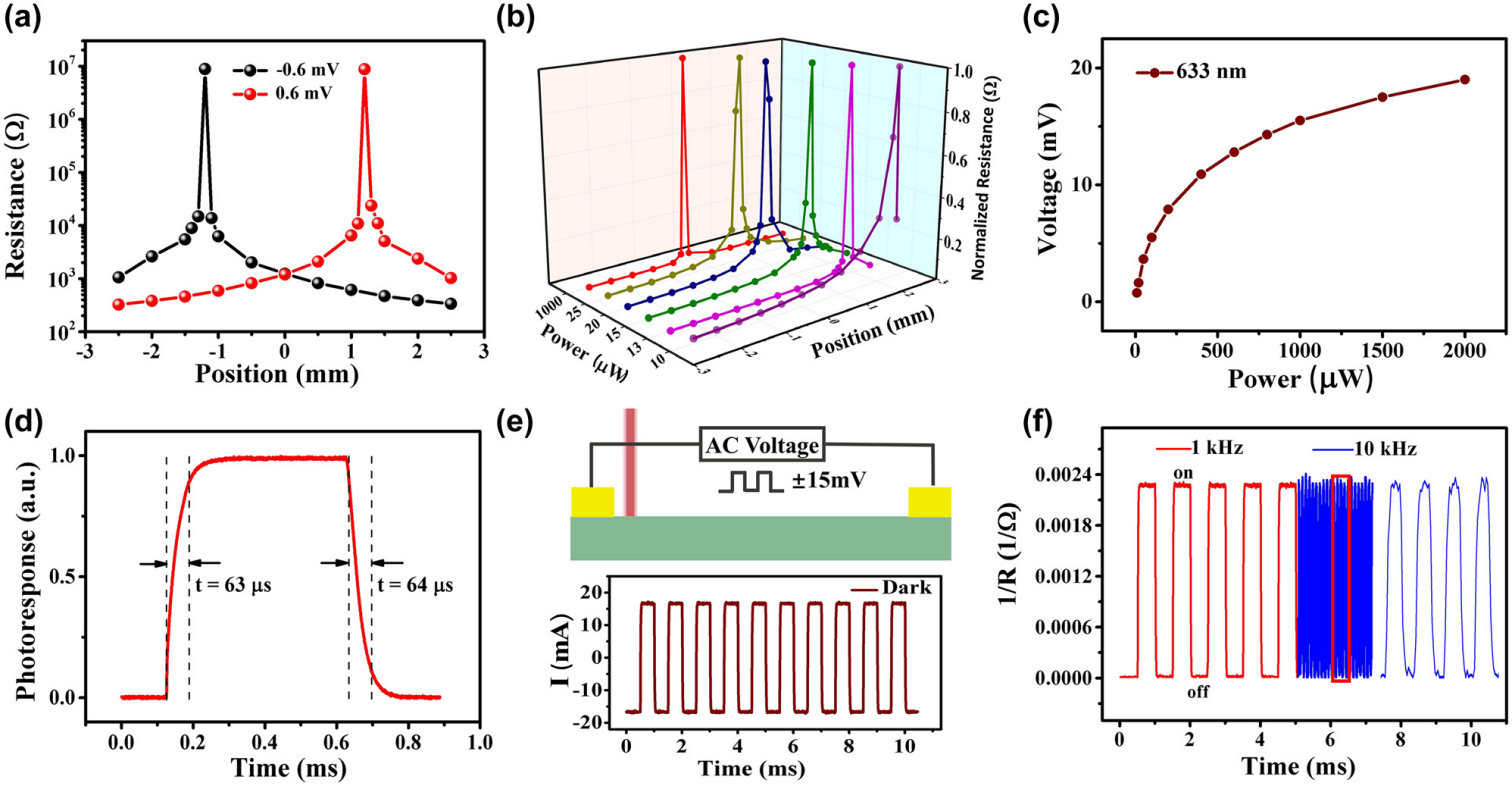
Position-dependent characteristics at small bias and high-frequency characteristics of devices. (a) The laser position dependence of device resistance under ±0.6 mV. (b) Dependence of device resistance and laser position at different powers. (c) The critical bias that produces the sudden resistance as a function of laser power. (d) Transient photoresponse of the device, showing that the rise time and fall time are 63 μs and 64 μs. (e) High frequency response characteristics of the device to AC voltage signal in the dark. (f) High frequency response characteristics under light irradiation. The right blue curve is the high frequency response curve of 10 kHz in the red box.

According to Equation (5), if *V*_*x*_ is comparable to the applied bias voltage, a sudden change in resistance occurs, which is verified experimentally. As shown in Figure 3a, the position-dependent resistance at 0.6 mV is performed under 633 nm light. The resistance exhibits 5 orders of magnitude change within the submillimeter range. Interestingly, if the bias voltage is negative (−0.6 mV), the resistance mutation position appears in the opposite direction of the symmetric position, also showing the bipolar resistance characteristics. Figure 3b shows the resistance mutation characteristics under different laser power. With the increase of power, the resistance mutation point gradually moves to the center. Even under very high laser powers, the resistance extreme point still lingers in the center position, which is consistent with the predictions from Equation (6). The threshold bias of resistance mutation under different power was obtained experimentally and plotted in Figure 3c. The voltage is found to gradually saturate as the power is increased, which is consistent with the phenomenon of electron–hole pairs saturation separation under high power [29]. In addition, the relationship between the resistance sudden change characteristic and the bias is also studied. As shown in the Figure S4, the mutation position gradually shifts from the electrode to the center as the applied bias is decreased, which is also in good agreement with the theoretical model.

**Figure 4:**
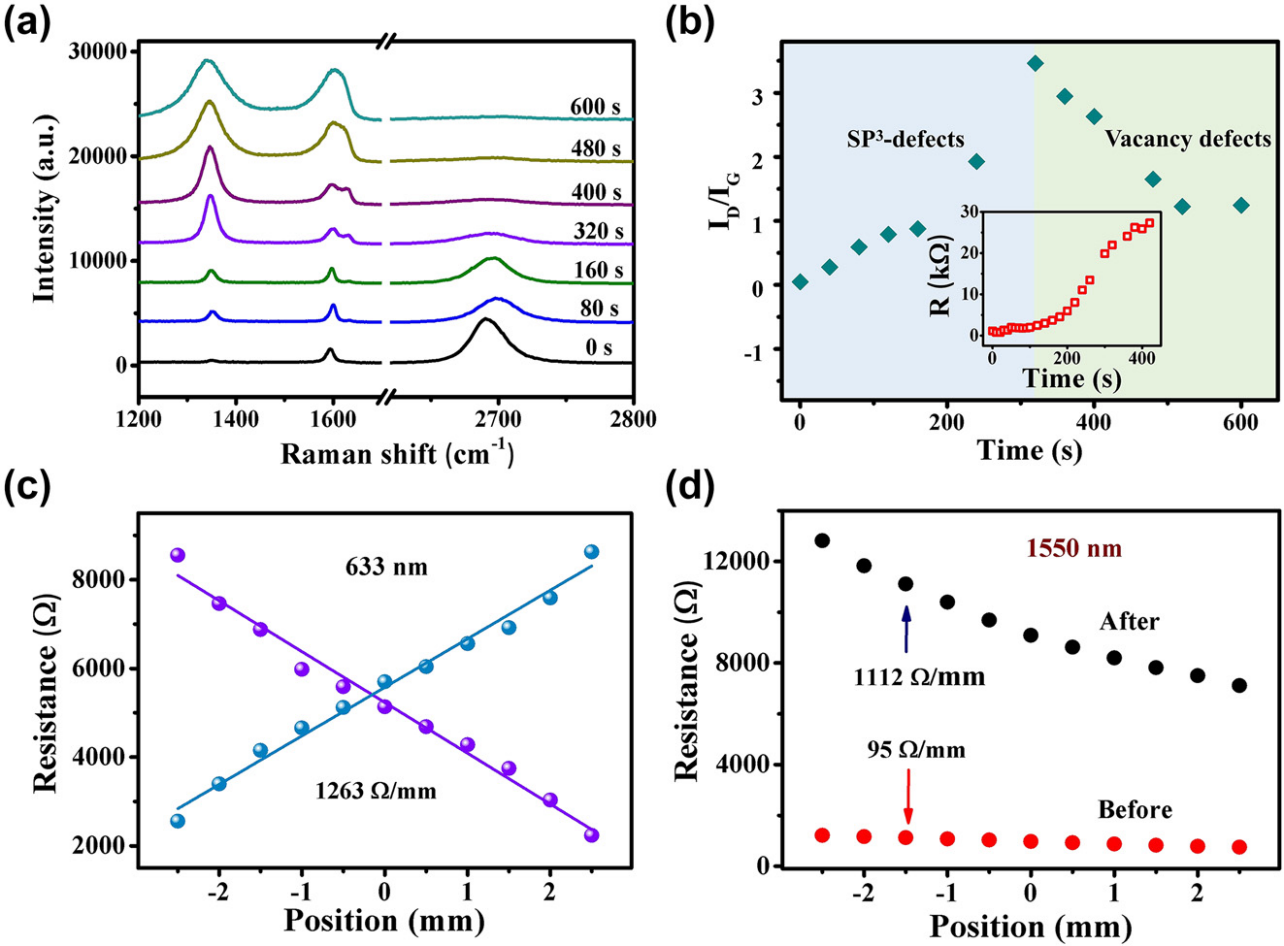
Plasma modification improves device performance. (a) Raman spectra of graphene under different oxygen plasma treatment times. (b) The variation of ratio *I*(*D*)/*I*(*G*) with increasing treatment time shows two distinct stages, suggesting the evolution of defect types. The inset shows the resistance of graphene under various oxygen plasma treatment durations. (c) The position dependence of resistance of plasma-modified devices under 633 nm laser irradiation. (d) The position-dependent characteristics of device resistance before and after plasma modification under 1,550 nm laser irradiation, where the resistance sensitivity can be seen to be improved by ∼11 times.

As can be seen from Figure 3a, the resistance of the device can span several orders of magnitude, which has a good application prospect in the field of optical switching. High frequency response characteristics of the device as an optical switch are investigated by controlling incident light and AC electrical signals. From the transient light response characteristic shown in Figure 4d, the rise time and fall time are calculated to be 63 μs and 64 μs, respectively, by intercepting the time difference of 10 % – 90 % of the photoresponse. High symmetry also implies high quality of the device. Figure 3e shows the response characteristics of AC voltage (±0.6 mV) with a frequency of 1 kHz in the dark. It can be seen that the current changes symmetrically and the resistance remains basically unchanged. When the light is irradiated at the critical point (Figure 3f), the current of the device under different polar voltages shows an order of magnitude difference, which means that the device switches between HRS and LRS at high speed. Benefiting from the high mobility of graphene, the device can respond to signals up to 10 kHz, demonstrating fast and reliable on-off switching performance, which is superior to the performance of conventional photoresistors.

In order to improve the application potential, the performance of the SDPR in the linear modulation region is further managed by taking advantage of the easy regulation of the two-dimensional material. Oxygen plasma is employed to modify the device surface, which can introduce functional groups or defects into the graphene and increase its resistance and sensitivity [37]. Figure 4a shows the evolution of Raman spectra of graphene on SiO_2_ substrate under plasma treatment. D peaks and D′ peaks representing defects in the Raman spectra gradually appear and increase with treatment time, indicating that large amounts of defects are introduced into graphene [29]. These defects mainly include vacancy defects, oxygen-containing functional groups, oxygen adsorption, etc. Defects in graphene can potentially suppress the characteristic lattice vibration mode associated with the 2D peak [37]. The gradual broadening and weakening of the 2D peak is also indicative of the evolution of graphene. Figure 4b shows ratio of ID/IG as a function of processing time, showing two distinct stages, which is consistent with previous reports [38], [39]. The defects in graphene are first dominated by sp^3^ defects, which then gradually convert to vacancy defects, and finally to amorphous carbon. XPS spectra (Figure S5 and Table S1) also indicates that graphene is more oxidized after plasma treatment but is still dominated by C–C sp^2^ bonds in our samples, rather than being converted to graphene oxide [40]. The introduction of defects will affect the electrical transport properties of graphene [41], as illustrated in Figure S6, where P-doping is significantly enhanced with increasing oxygen plasma treatment time. Simultaneously, the mobility of graphene is degraded due to the defects acting as scattering centers, consequently elevating the resistance of the device. Figure 4c displays the bipolar resistance characteristics of the plasma-treated SDPR. It is evident that the resistance sensitivity post-surface modification increase to 1,263 Ω/mm at visible wavelengths (633 nm) and maintains long-term stability (Figure S7). In addition, the modulation of position-sensitive resistance characteristics in the infrared band is also investigated. Figure 4d shows the resistance characteristics under 1,550 nm laser before and after plasma processing, where the resistance sensitivity is found to increase from 95 Ω/mm to 1,112 Ω/mm, exhibiting a near 11-fold improvement. This high-performance broadband photo-rheostat has great application potential in logic devices and memory devices.

## 3 Conclusions

In summary, a position-dependent photo-rheostat is reported that is based on a graphene–Si heterojunction. The resistive sensitivity of up to 240 Ω/mm is obtained in the linear regulation region, which is further improved to 1,263 Ω/mm by oxygen plasma treatment modification. The device further achieves resistance modulation of 5 orders of magnitude under very low power consumption. A model is established a model from the perspective of carrier dynamics to reveal the mechanism of the abrupt resistance change, which agrees well with our obtained data. Benefiting from the high mobility of graphene, such a heterojunction device exhibits a rapid photoresponse ∼63 µs that enables an operating frequency up to 10 kHz for switching. In addition, using the combined absorption of Si and graphene, the device can operate at wavelengths covering the visible-near infrared (1,550 nm). The proposed position-dependent photo-rheostat device offers the advantage of being ultra-fast, broadband, and consumes low power, which holds great potential in the field of photoelectric logic switching applications.

## 4 Methods

### 4.1 Preparation of the device

The graphene employed in the device is monolayer graphene sample grown on copper foil by chemical vapor deposition (CVD) [42]. The graphene film attached to PMMA was obtained by etching in 0.1 M ammonium persulfate solution and washing in deionized water several times. N-type Si substrate with 1–10 Ω cm resistivity was first soaked in 3 % HF for 2 min to remove surface impurities and oxides and then stood in clean air for 2 h to obtain a uniform natural oxide layer. The graphene film was transferred to the Si substrate by wet transfer and then heated in a vacuum at 250 °C for 1 h to bind the two more tightly. The heterojunction samples were then immersed in acetone to remove PMMA on graphene. The electrodes (5 nm Ni/50 nm Au), which are 5 mm apart, were deposited on the surface by using mask and thermal evaporation to complete the device preparation.

### 4.2 Device characterization

The measurements of the devices were carried out at room temperature under atmospheric atmosphere. A semiconductor analyzer FS-Pro was employed to characterize all electrical characteristics. The position-dependent response characteristics were achieved by moving the device using an electric displacement platform. A 633 nm semiconductor laser and a continuously adjustable (680–1,600 nm) femtosecond laser (Chameleon Ultra II) were used as light sources. For high frequency response measurement, a signal generator (DG4162) was used to provide high frequency AC signals, and an acoustic optical modulator (R21080-1DS) was used to modulate laser at high speed to measure photoresponse speed of the device. In addition, a Raman spectrometer (Horiba HR-800) was used to record the Raman spectra of the sample.

## Supplementary Material

Supplementary Material Details
