# Supplementary material for: Fast and broadband spatial-photoresistance modulation in graphene–silicon heterojunctions
Source: Nanophotonics. 2024 Jun 17;13(19):3663–70. doi: 10.1515/nanoph-2024-0084 (PMC11465980; doi:10.1515/nanoph-2024-0084)
Supplement: Supplementary file 1 — Supplementary Material Details [file j_nanoph-2024-0084_suppl_001.docx]

**Supporting information**

Ruxia Du^#^, Wenhui Wang^#^, Huiwen Lin, Xinlei Zhang, Hao Wu, Beibei Zhu, Xu Jing, Xing Gu, Zhenhua Ni^[[1]](#footnote-0)^, Li Tao^1^

**Fast and Broadband Spatial-Photoresistance Modulation in Graphene-Silicon Heterojunctions**


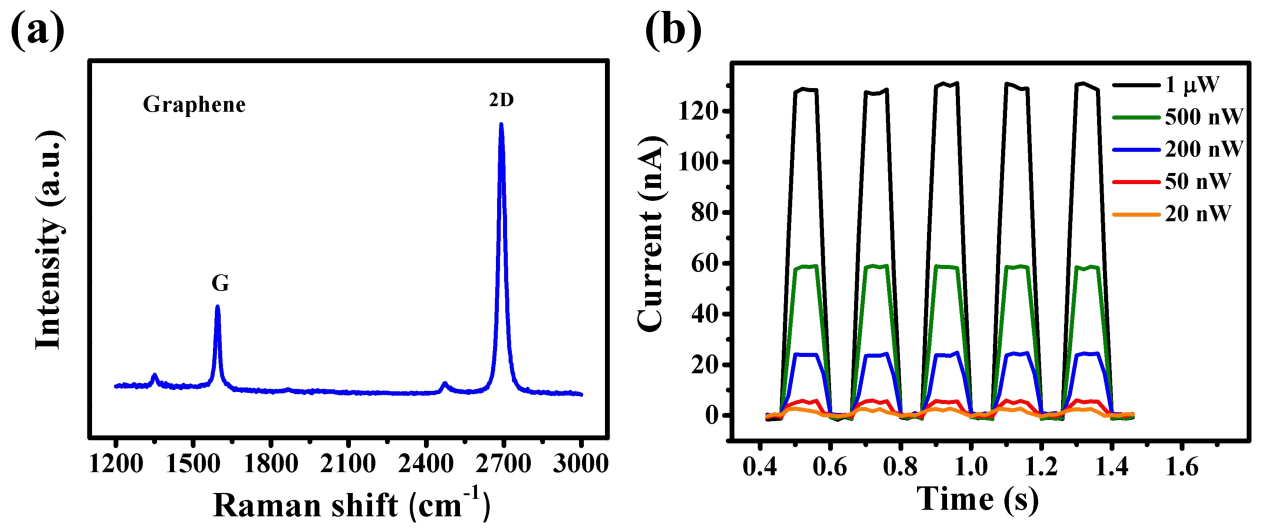


**Figure S1**. (a) Raman spectrum of the CVD graphene. (b) Photoresponse characteristics of the graphene-Si heterojunction under varying light power demonstrating the consistent weak photoresponse of the device.


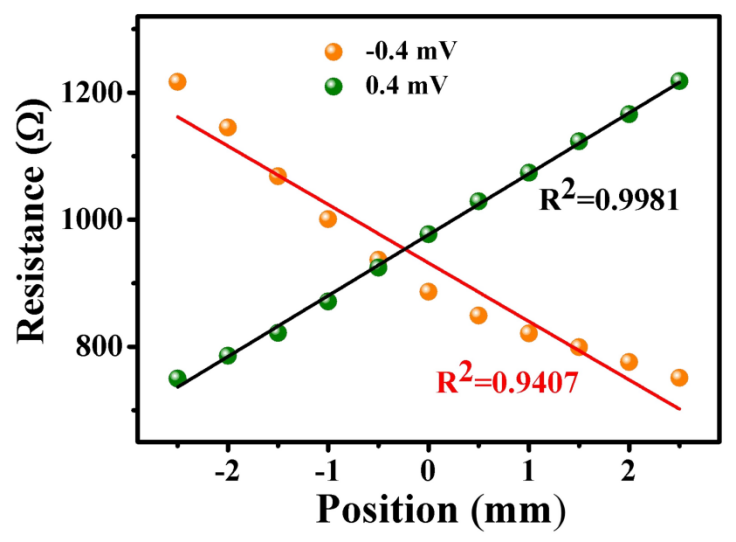


**Figure S2.** Position dependence of resistance under 1550 nm laser irradiation and ±0.4 mV bias voltage.


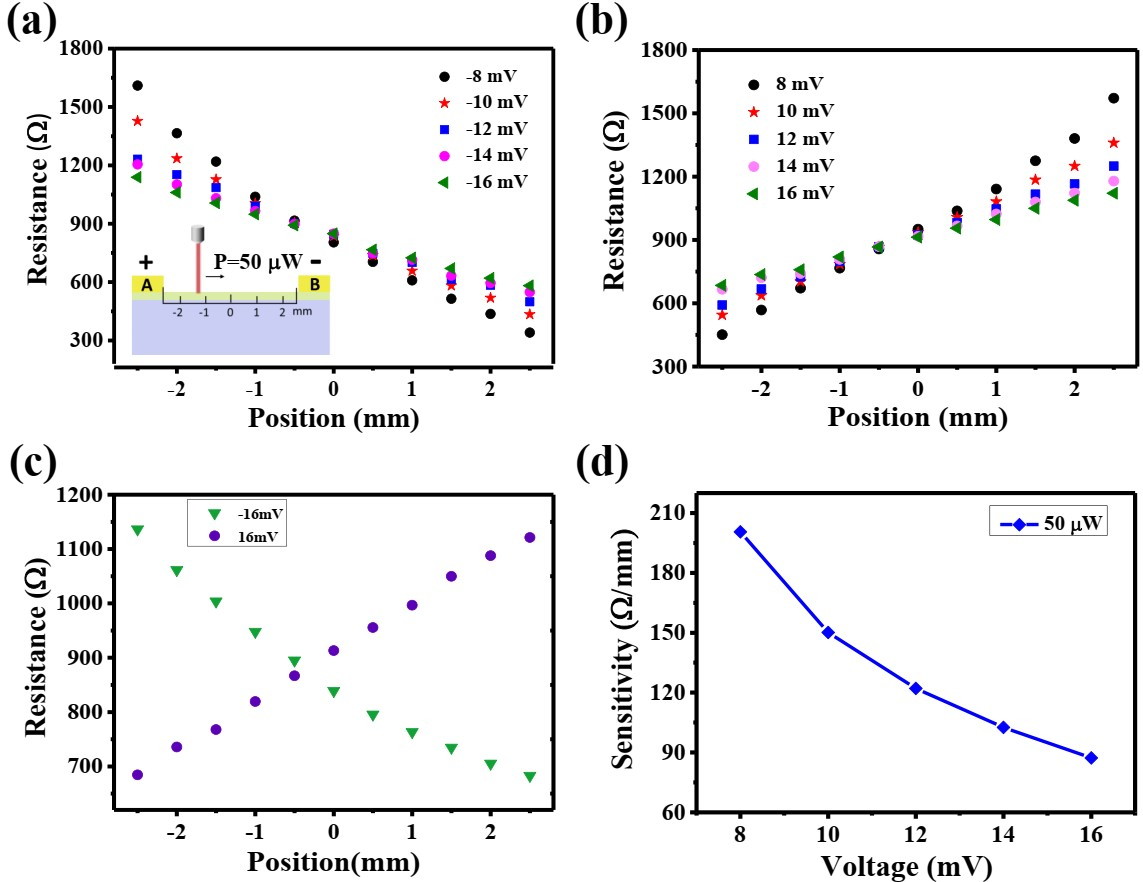


**Figure S3.** The laser position dependence of resistance at various positive (a) and negative (b) bias voltages. The inset shows the schematic diagram of device operation. (c) Position dependence of device resistance under ±16 mV bias voltage. (d) The dependence of resistance sensitivity with applied bias.


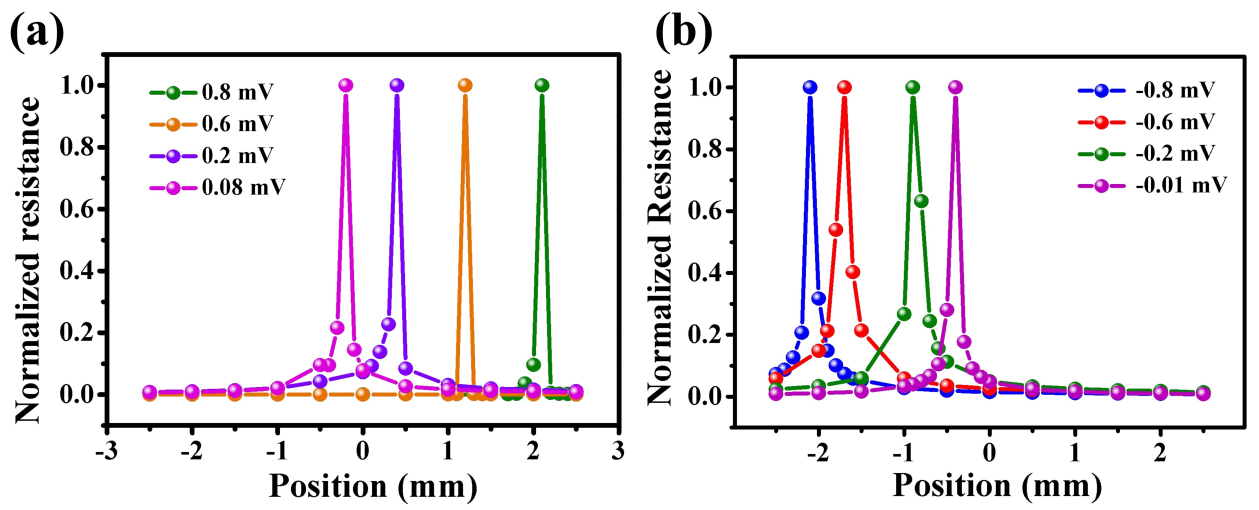


**Figure S4.** The laser position dependence of normalized resistance under varying positive (a) and negative (b) bias voltages.


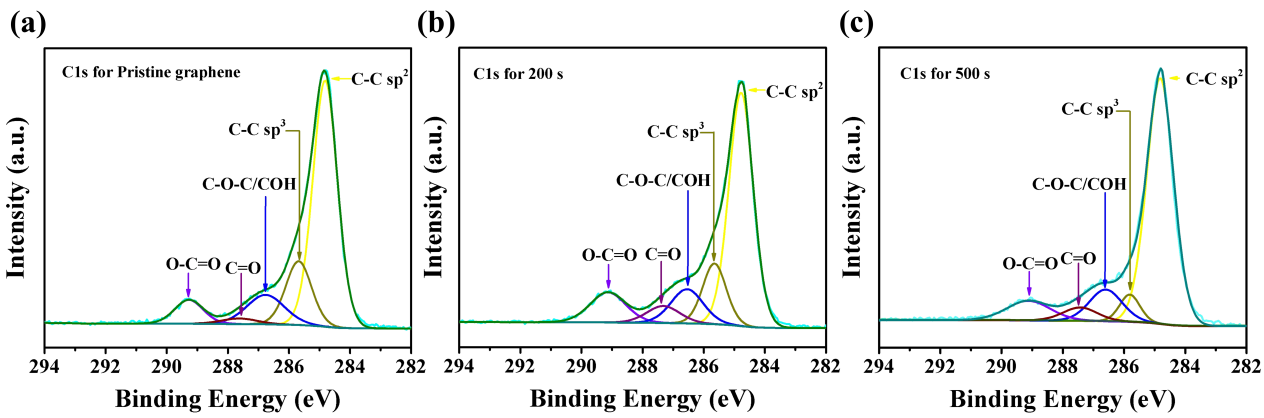


**Figure S5.** Typical high-resolution XPS spectra in C1s region of pristine graphene (a) and graphene treated with oxygen plasma for 200 s (b) and 500 s (c).

**Table S1.** XPS data of pristine graphene and graphene treated with oxygen plasma for 200 s and 500 s.

**
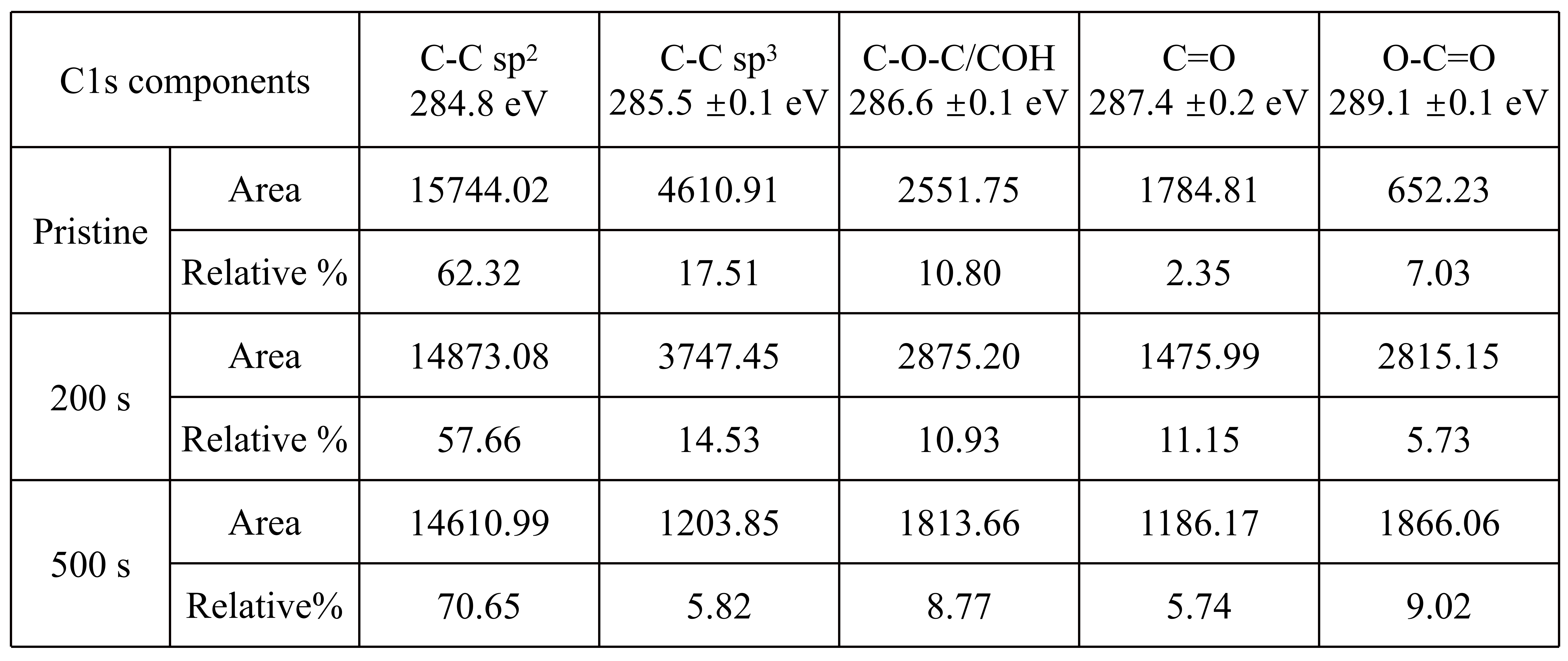
**

After oxygen plasma treatment, graphene will develop defects, with some of them potentially absorbing oxygen or containing oxygen functional groups. We have measured XPS spectra of pristine graphene and graphene treated with oxygen plasma for 200 s and 500 s. As shown in Figure S5 and Table S1, the collected signals can be fitted to five peaks with binding energies near 284.8, 285.5, 286.6, 287.4, 289.1 eV, which are attributed to sp^2^ C, sp^3^ C, C-O-C/COH, C=O, O-C=O bonds, respectively. It can be seen that XPS spectra of three samples are all dominated by C-C sp^2^ bonds, indicating that graphene has not been converted to graphene oxide after oxygen plasma treatment. However, the degree of oxygenated functionalization increased after plasma treatment, because the compositional ratio of the three oxygen-containing groups (C-O-C/COH, C=O, O-C=O) are increased from 20.18% (0 s) to 27.81% (200 s) and then to 23.53% (500 s). These non-linear variations in chemical bond ratio align with alterations in the type of defects induced by oxygen plasma treatment of graphene, which is in accordance with Raman result (Figure 4(b)).


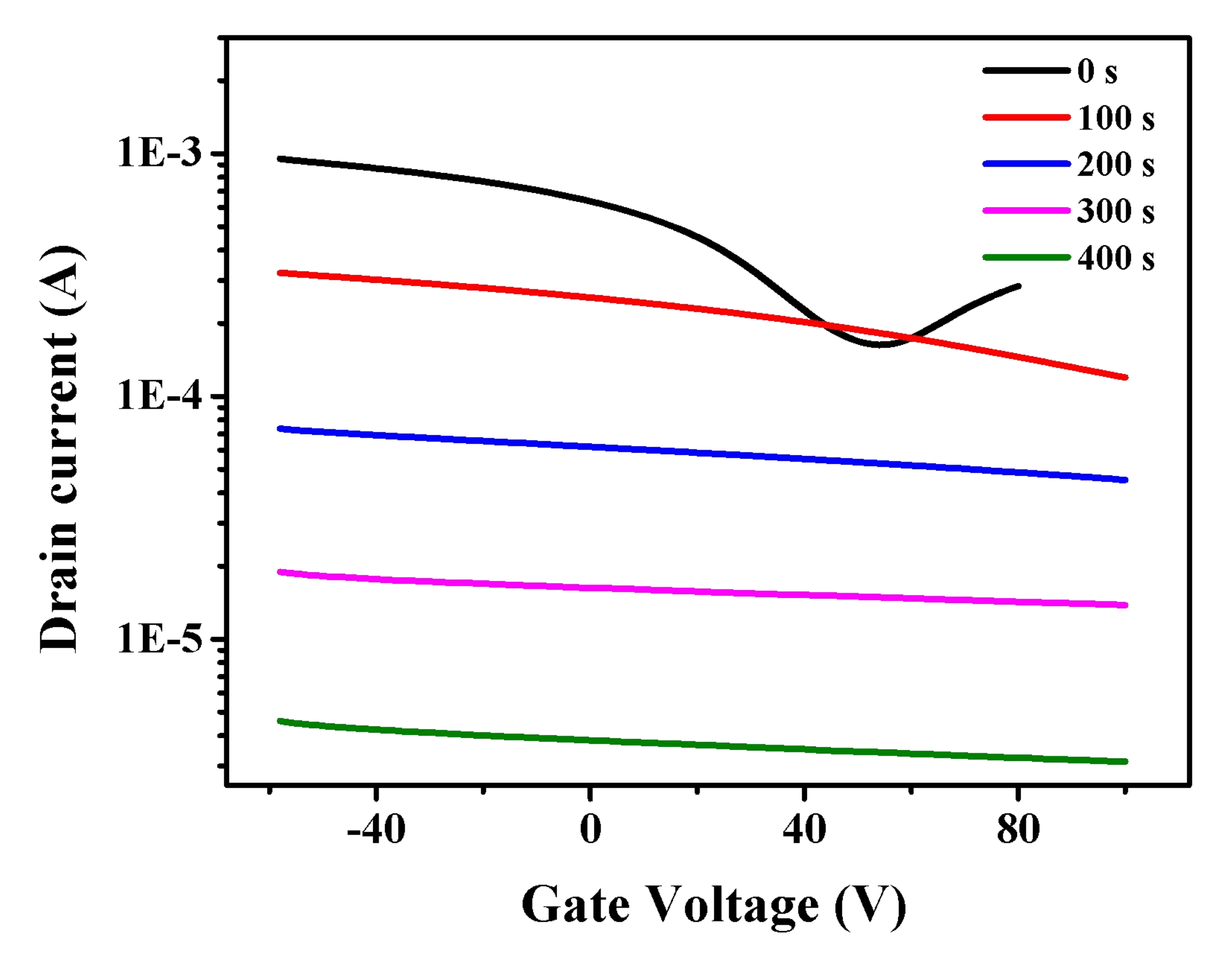


**Figure S6.** Transfer curves of graphene under different oxygen plasma treatment times.

As depicted in the Figure S6, the pristine graphene exhibits P-type doping, while oxygen plasma treatment results in significant enhancement of P-type doping and degradation of the current (i.e., an increase in resistance). This can be attributed to the introduction of defects in graphene, such as oxygen-containing functional groups and vacancies, through oxygen plasma treatment. These defects act as scattering centers during electrical transport, leading to a decrease in mobility and an increase in the resistance of graphene.


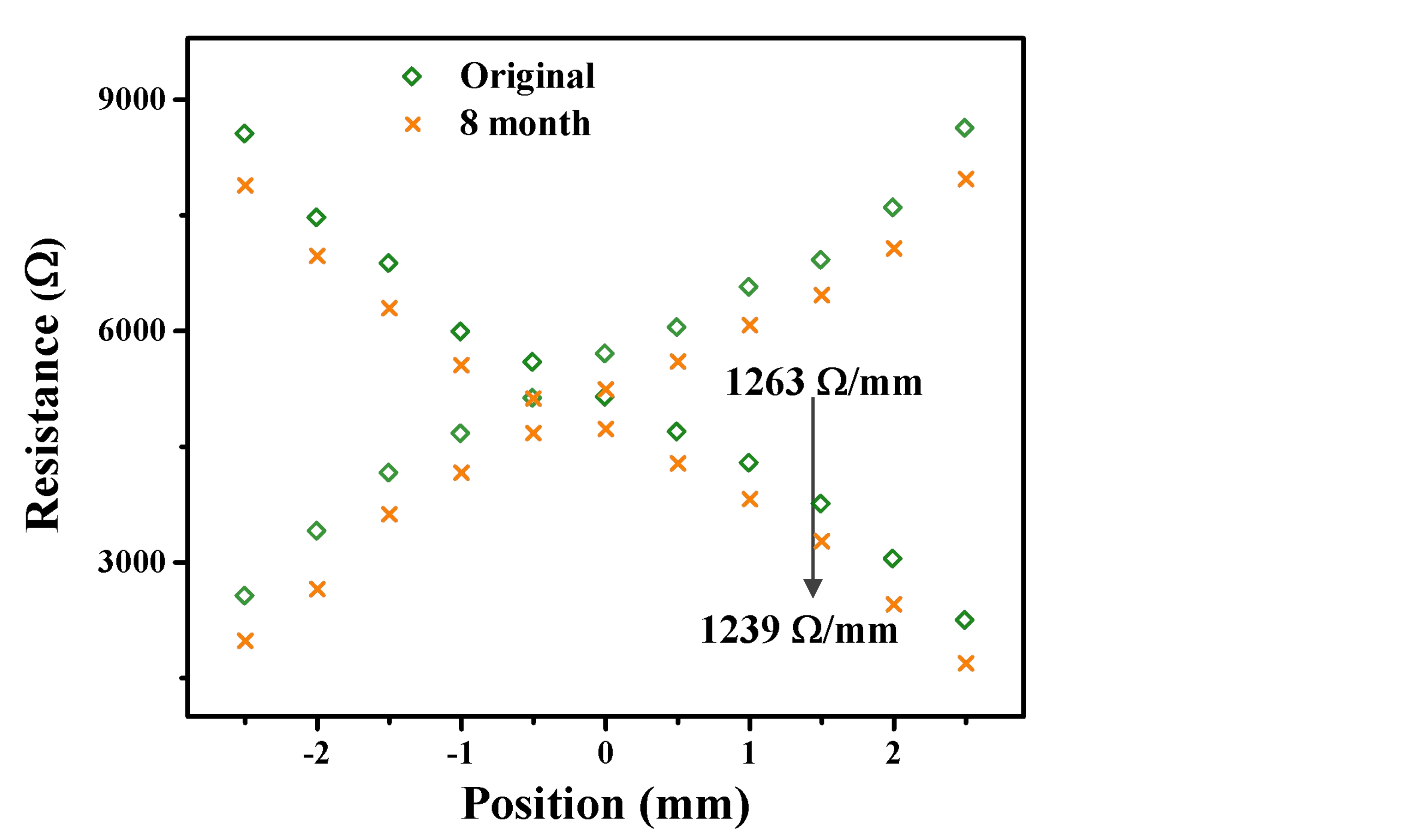


**Figure S7.** Stability of the position dependence of resistance of graphene-Si device stored in ambient environment for 8 months.

1. ^#^ Ruxia Du and Wenhui Wang contributed equally to this work.

   **Corresponding authors:** **Zhenhua Ni,** Key Laboratory of Quantum Materials and Devices of Ministry of Education, School of Physics, Southeast University, Nanjing 211189, P.R. China, **Li Tao,** Key Laboratory of Quantum Materials and Devices of Ministry of Education, School of Materials Science and Engineering, E-mail: [tao@seu.edu.cn](mailto:tao@seu.edu.cn)

   **Ruxia Du, Huiwen Lin, Beibei Zhu, Xu Jing and Xing Gu,** School of Materials Science and Engineering, Southeast University, Nanjing 211189, P.R. China, E-mail: [durx@seu.edu.cn](mailto:durx@seu.edu.cn) (R. Du), [linh@seu.edu.cn (H](mailto:linh@seu.edu.cn%20(H). Lin), [101012333@seu.edu.cn (B](mailto:101012333@seu.edu.cn%20(B), Zhu), [xjing@seu.edu.cn (X](mailto:xjing@seu.edu.cn%20(X). Jing), [xinggu@seu.edu.cn (X](mailto:xinggu@seu.edu.cn%20(X). Gu)

   **Wenhui Wang, Xinlei Zhang and Hao Wu,** Key Laboratory of Quantum Materials and Devices of Ministry of Education, School of Physics, Southeast University, Nanjing 211189, P.R. China, E-mail: [wangwh@seu.edu.cn (W. Wang)](mailto:wangwh@seu.edu.cn%20(W.%20W)), [zhangxinlei505080@163.com (X. Z](mailto:zhangxinlei505080@163.com%20(Z)hang), [20222238@seu.edu.cn](mailto:20222238@seu.edu.cn) (H. Wu) [↑](#footnote-ref-0)
